# ECF Sigma Factor HxuI Is Critical for *In Vivo* Fitness of *Pseudomonas aeruginosa* during Infection

**DOI:** 10.1128/spectrum.01620-21

**Published:** 2022-01-19

**Authors:** Zeqiong Cai, Fan Yang, Xiaolong Shao, Zhuo Yue, Zhenpeng Li, Yuqin Song, Xiaolei Pan, Yongxin Jin, Zhihui Cheng, Un-Hwan Ha, Jie Feng, Liang Yang, Xin Deng, Weihui Wu, Fang Bai

**Affiliations:** a State Key Laboratory of Medicinal Chemical Biology, Key Laboratory of Molecular Microbiology and Technology of the Ministry of Education, College of Life Sciences, Nankai University, Tianjin, China; b Department of Biomedical Sciences, City University of Hong Kong, Kowloon Tong, Hong Kong SAR, China; c School of Laboratory Medicine, Key Laboratory of Clinical Laboratory Diagnostics in Universities of Shandong, Weifang Medical University, Weifang, Shandong, China; d State Key Laboratory of Microbial Resources, Institute of Microbiology, Chinese Academy of Sciences, Beijing, China; e Department of Biotechnology and Bioinformatics, Korea University, Sejong, Republic of Korea; f School of Medicine, Southern University of Science and Technology (SUSTec), Shenzhen, China; Emory University School of Medicine

**Keywords:** *Pseudomonas aeruginosa*, ECF sigma factor, HxuI, host stress-response, virulence

## Abstract

The opportunistic pathogen Pseudomonas aeruginosa often adapts to its host environment and causes recurrent nosocomial infections. The extracytoplasmic function (ECF) sigma factor enables bacteria to alter their gene expression in response to host environmental stimuli. Here, we report an ECF sigma factor, HxuI, which is rapidly induced once P. aeruginosa encounters the host. Host stresses such as iron limitation, oxidative stress, low oxygen, and nitric oxide induce the expression of *hxuI*. By combining RNA-seq and promoter-*lacZ* reporter fusion analysis, we reveal that HxuI can activate the expression of diverse metabolic and virulence pathways which are critical to P. aeruginosa infections, including iron acquisition, denitrification, pyocyanin synthesis, and bacteriocin production. Most importantly, overexpression of the *hxuI* in the laboratory strain PAO1 promotes its colonization in both murine lung and subcutaneous infections. Together, our findings show that HxuI, a key player in host stress-response, controls the *in vivo* adaptability and virulence of P. aeruginosa during infection.

**IMPORTANCE** P. aeruginosa has a strong ability to adapt to diverse environments, making it capable of causing recurrent and multisite infections in clinics. Understanding host adaptive mechanisms plays an important guiding role in the development of new anti-infective agents. Here, we demonstrate that an ECFσ factor of P. aeruginosa response to the host-inflicted stresses, which promotes the bacterial *in vivo* fitness and pathogenicity. Furthermore, our findings may help explain the emergence of highly transmissible strains of P. aeruginosa and the acute exacerbations during chronic infections.

## INTRODUCTION

Pseudomonas aeruginosa is a Gram-negative opportunistic pathogen that causes various health care-associated infections, including pneumonia, burn wound infections, sepsis, urinary tract infections, and surgical site infections (1–3). To establish an effective infection, pathogens have to contend with host-inflicted stresses, such as iron deprivation (4), hypoxia (5), oxidative stress (1), and nitrosative stress (6, 7). The cell-surface signaling (CSS) system is a membrane-spanning signaling pathway that allows Gram-negative bacteria to transduce extracellular stimuli into coordinated transcriptional responses, and thus plays an important role in regulating bacterial adaptability and pathogenicity in response to diverse niches (8).

Typically, the CSS system is a tripartite molecular device that is composed of (i) an outer membrane TonB-dependent receptor, which senses the extracellular stimulus; (ii) a cytoplasmic membrane-spanning anti-σ factor involved in signal transduction from the periplasm to the cytoplasm; and (iii) an extracytoplasmic function (ECF) sigma factor that initiates transcription by directing core RNA polymerase (cRNAP) to the stimulus-responsive target gene(s) (8). The ECFσ family is highly diverse, and a comprehensive classification has been reported based on more than 2,700 ECFσ from hundreds of bacterial genomes (9). These ECFσ often act orthogonally with limited cross talk and allow the partitioning of the transcriptional space. The high stringency of ECFσ promoter recognition restricts the number of target genes to mount specific responses (10). In P. aeruginosa, the strains PAO1 and PA14 encode 19 and 21 ECFσ factors, respectively. They mediate the functions of cell envelope stress response, production of the exopolysaccharide alginate, iron uptake, and pathogenicity (8, 11).

**FIG 1:**
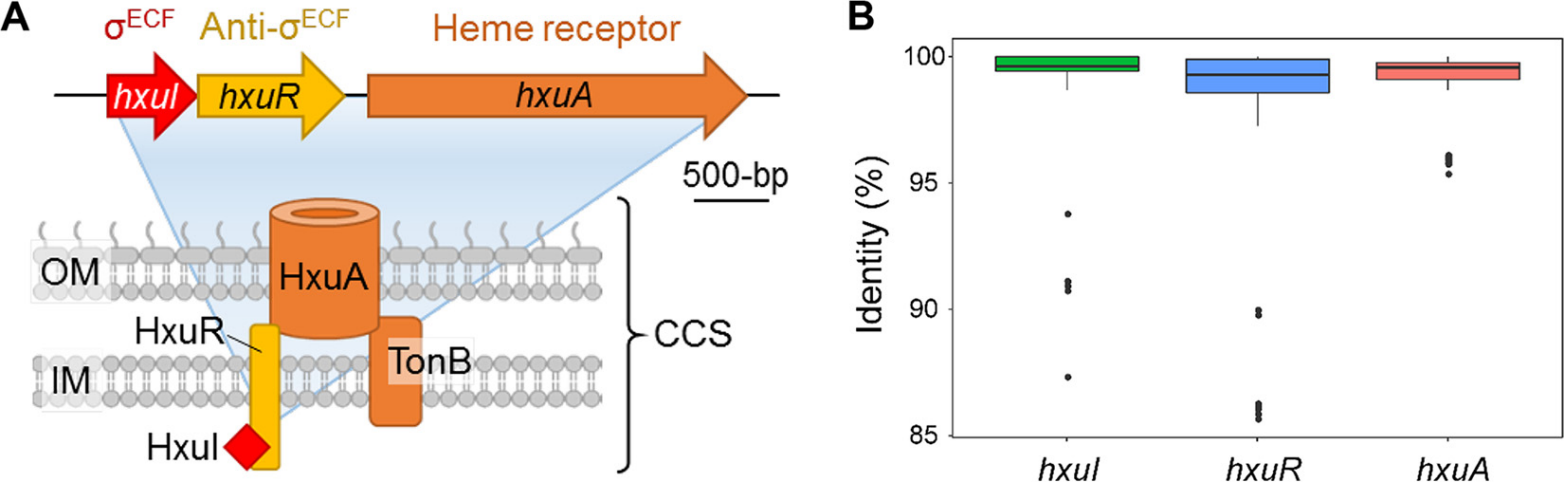
Conservation of Hxu system in P. aeruginosa. (A) Schematic representation of Hxu system. (B) Conservation analysis of *hxuAIR* genes in 723 P. aeruginosa strains. Dots represent outliers from the respective groups.

The Hxu CSS pathway, which consists of three adjacent genes *hxuIRA* encoding ECFσ factor, anti-σ factor, and TonB-dependent outer membrane receptor, respectively (Fig. 1A), has been recently shown to be involved in heme signaling in P. aeruginosa, and mediates heme acquisition from host hemopexin (12, 13). However, the target genes of the ECFσ factor HxuI remain unknown. In the present study, we found that HxuI is highly conserved in different P. aeruginosa strains. In addition to heme, the host stresses of iron limitation, oxidative stress, hypoxia, and nitric oxide can all induce the expression of HxuI which, in turn, controls a variety of physiological functions associated with P. aeruginosa infection, including iron acquisition, anaerobic respiration, pyocyanin synthesis, and pyocin production. Most importantly, overexpression of *hxuI* in PAO1 promoted bacterial colonization and long-term infection in various murine infection models. Together, these studies suggest that HxuI is an important ECFσ factor contributing to the *in vivo* fitness and pathogenicity of P. aeruginosa.

## RESULTS

### HxuI is highly conserved in *P. aeruginosa*.

To analyze the conservation of the Hxu system, 723 P. aeruginosa clinical isolates with available genome sequences were analyzed by BLASTn (14). All strains possessed the *huxIRA* genes, and the *hxuI* gene is highly conserved among various P. aeruginosa strains (Fig. 1B), reflecting its important physiological functions.

### Host stresses induce the expression of ECFσ factor HxuI.

**FIG 2:**
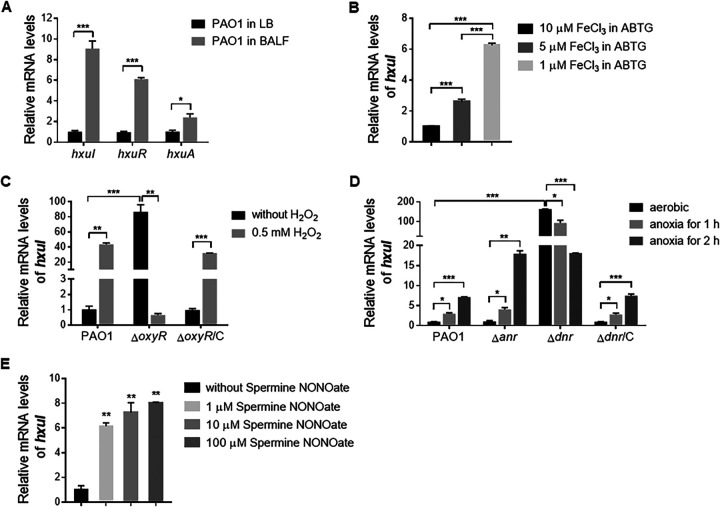
Host stresses-response of ECFσ HxuI. (A) Mice were infected with 1 × 10^7^ CFU of PAO1 intranasally. BALF was harvested from 16 mice at 6 h postinfection and pooled for bacterial cell isolation and subsequent RNA purification. Relative mRNA levels of *hxuIRA* genes of PAO1 in mouse BALF and LB medium were measured by qPCR. (B to E) qPCR determination of *hxuI* expression levels in wild-type PAO1, mutants, and the complemented strains (Δ*oxyR*/C and Δ*dnr*/C) under conditions of Fe(III) limitation (panel B), hydrogen peroxide exposure (panel C), hypoxia (panel D), and NO donor Spermine NONOate treatment (panel E). Housekeeping gene *ppiD* was used as the internal reference. Error bars represent SD. *, *P* < 0.05; **, *P* < 0.01; ***, *P* < 0.001.

To test whether Hxu responds to the host environment during infection, we infected mice with a laboratory strain PAO1 intranasally and collected bacterial cells from the bronchoalveolar lavage fluid (BALF) 6 h postinfection (pi). Quantitative real-time PCR (qPCR) assays showed that the *hxuIRA* genes were upregulated 9-, 6.1-, and 2.4-fold, respectively (Fig. 2A), indicating that Hxu indeed responds to the host environment. To address the *in vivo* inducing signals, we tested a number of well-known host stress conditions to determine their effects on *huxI* gene expression. First, we tested *hxuI* expression under iron-deficient conditions. In the PAO1 strain, *hxuI* expression was increased with the decrease of Fe(III) in ABTG medium (Fig. 2B). During host infection, phagocytic cells generate reactive oxygen species (ROS) such as superoxides, which are involved in antibacterial activity (15). Next, we tested *hxuI* expression under oxidative stress. In the wild-type (wt) PAO1 strain, exposure to 0.5 mM H_2_O_2_ for 30 min induced a 43-fold increase in *hxuI* expression (Fig. 2C). OxyR is an H_2_O_2_-responsive regulator which activates the expression of defense genes against oxidative stress in P. aeruginosa (16). In an *oxyR* mutant, the expression of *hxuI* increased 86-fold even without the H_2_O_2_ treatment, indicating a repressive effect of OxyR on *hxuI* expression, which was restored by complementation with the *oxyR* gene (Fig. 2C). Possible explanations revolve around the significant regulatory cross-talk in the management of redox-stress and iron homeostasis through ferric uptake regulator (Fur) (17). Nonetheless, these data indicated that hydrogen peroxide induces the expression of HxuI in the presence of OxyR.

P. aeruginosa is able to grow in the absence of oxygen through anaerobic metabolism, which influences infectivity as well as biofilm formation (18). To investigate whether HxuI responds to hypoxia, we determined *hxuI* expression by qPCR after a short incubation under anaerobic conditions. As shown in Fig. 2D, *hxuI* expression increased along with the anaerobic culture time in the PAO1 strain. There are two well-known anaerobic sensors in P. aeruginosa: ANR and DNR (5). The expression of *hxuI* was increased under anaerobic growth conditions in an *anr* mutant, but not in a *dnr* mutant background (Fig. 2D). However, under aerobic conditions, *hxuI* expression increased by 164-fold in the *dnr* mutant but did not change in the *anr* mutant (Fig. 2D), indicating a negative regulation of *hxuI* by DNR. Complementation with a *dnr* gene restored *hxuI* expression levels in the Δ*dnr* mutant (Fig. 2D). Since DNR is known to sense nitric oxide (NO), and NO-dependent DNR activity requires heme (18), we further tested whether NO directly induces the expression of HxuI. When the PAO1 wt strain was treated with 1 to 100 μM NO donor Spermine NONOate (19) for 30 min, the expression of *hxuI* was increased significantly in a dose-dependent manner (Fig. 2E). The above data suggested that oxygen limitation, likely via NO, induces the expression of HxuI.

### Identification of the HxuI regulon genes.

**FIG 3:**
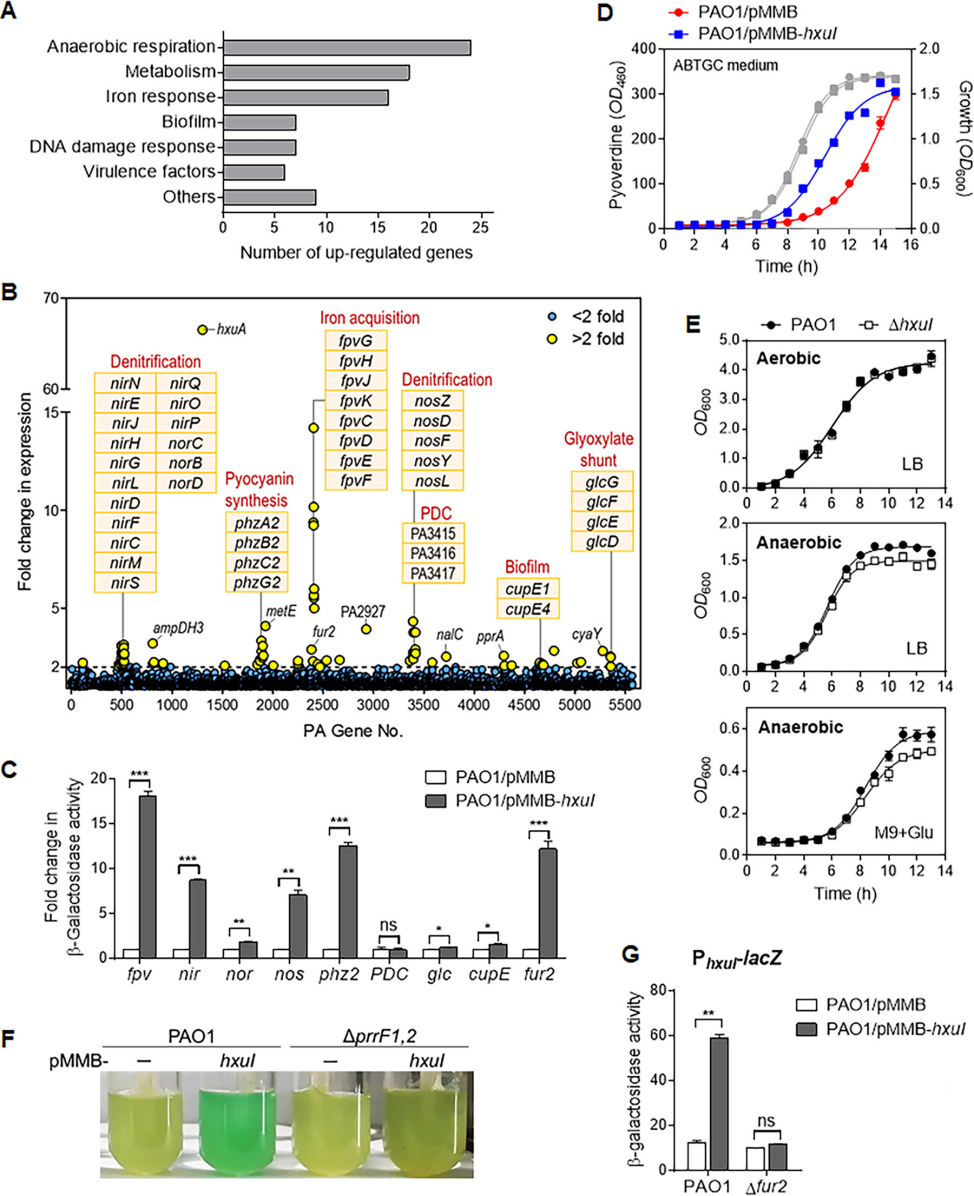
The ECFσ HxuI regulon in P. aeruginosa. (A) Functional classification of upregulated genes in RNA-seq of HxuI-overexpressing strain. (B) Gene clusters that were remarkably upregulated in HxuI overexpressor. PDC, pyruvate dehydrogenase complex. (C) Analysis of the promoter-*lacZ* receptor expression. P. aeruginosa PAO1 containing the indicated *lacZ* transcriptional fusions, the plasmid pMMB (empty plasmid), or the plasmid pMMB-*hxuI* were grown in LB with 1 mM IPTG until late exponential growth phase and analyzed for β-galactosidase activity. Fold changes compared to PAO1/pMMB are shown. (D) Pyoverdine production (blue and red curves) and growth curves (gray) of indicated strains. (E) Growth curves of wt PAO1 and *hxuI* mutant under aerobic or anaerobic conditions. Glu, glucose. (F) PAO1 containing empty vector pMMB or pMMB-*hxuI* were grown in iron-deficient medium with 1 mM IPTG until late exponential growth phase; the presence of the green pigment indicates pyocyanin production. (G) Expression of *hxuI* promoter-*lacZ* receptor fusion in PAO1 and *fur2* mutant backgrounds. Error bars represent SD. *, *P* < 0.05; **, *P* < 0.01; ***, *P* < 0.001; ns, not significant.

To gain insights into the HxuI regulons, a transcriptomic study was performed on P. aeruginosa PAO1 overexpressing the *hxuI* gene on an inducible expression vector pMMB. Most ECFσ are subject to positive auto-regulation and directly induce the expression of corresponding TonB receptor, thereby enhancing their signaling effect for as long as the inducing conditions prevail (11). The expression level of TonB receptor *hxuA* was monitored by qPCR at various isopropyl β-d-thiogalactopyranoside (IPTG) induction times, and it was found that *hxuA* expression peaked at 2 h postinduction (Fig. S1). Accordingly, total RNA samples of PAO1/pMMB-*hxuI* and PAO1/pMMB strains were collected 2 h after induction by 1 mM IPTG, and these were then subjected to RNA-seq analysis. The overexpression of *hxuI* resulted in the upregulation of 87 genes and the downregulation of 22 genes at rates of more than 2-fold (*P* value ≤ 0.05). Of the 87 genes significantly upregulated by HxuI, 24 genes are involved in anaerobic respiration and denitrifying redox chain, 18 are involved in metabolism, 16 in iron acquisition, 7 in biofilm formation, 7 in DNA damage response, and 6 in virulence (Fig. 3A and Table 1).

**TABLE 1:** Upregulated genes of P. aeruginosa PAO1 overexpressing the ECFσ factor HxuI

Locus tag	Gene	Description	Promoter region motif	Fold change^*a*^	*P* value
Anaerobic respiration					
PA0112		Hypothetical protein		2.2	6.13E-06
PA0113		Cytochrome C oxidase assembly factor		2.21	6.07E-10
PA0509	*nirN*	Cytochrome C	Anr box	2.61	4.52E-38
PA0510	*nirE*	Uroporphyrin-III C-methyltransferase	Anr box	2.67	3.9E-30
PA0511	*nirJ*	Heme d1 biosynthesis protein	Anr box	2.32	1.18E-28
PA0512	*nirH*	Heme d1 biosynthesis protein	Anr box	2.25	2.72E-31
PA0513	*nirG*	Heme d1 biosynthesis protein	Anr box	2.05	3.07E-19
PA0515	*nirD*	Heme d1 biosynthesis protein	Anr box	2.05	1.85E-19
PA0516	*nirF*	Heme d1 biosynthesis protein	Anr box	2.13	2.79E-20
PA0518	*nirM*	Cytochrome C-551	Anr box	2.96	NA
PA0519	*nirS*	Nitrite reductase	Anr box	2.97	2.59E-20
PA0520	*nirQ*	Denitrification regulatory protein	Anr box	3.13	2.71E-08
PA0521	*nirO*	Cytochrome C oxidase subunit	Anr box	2.98	2.93E-07
PA0522	*nirP*	Hypothetical protein	Anr box	2.18	0.000407
PA0523	*norC*	Nitric oxide reductase subunit C	Dnr binding site	2.29	0.000129
PA0524	*norB*	Nitric oxide reductase subunit B	Dnr binding site	2.61	1.04E-05
PA0525	*norD*	Denitrification protein	Dnr binding site	2.68	8.35E-06
PA1847	*nfuA*	Fe/S biogenesis protein		2.12	1.74E-10
PA3392	*nosZ*	Nitrous-oxide reductase	Dnr box	4.32	2.49E-39
PA3393	*nosD*	Copper-binding periplasmic protein	Dnr box	2.74	1.7E-31
PA3394	*nosF*	Copper ABC transporter ATP-binding protein	Dnr box	2.57	5.34E-27
PA3395	*nosY*	Membrane protein	Dnr box	2.41	8.01E-16
PA3396	*nosL*	Accessory protein	Dnr box	3.77	4.13E-14
PA5275	*cyaY*	Frataxin-like protein; iron-sulfur cluster assembly protein		2.82	1.26E-25

Metabolism					
PA0494		Probable acyl-CoA carboxylase (ACCase) subunit		2.44	0.0000198
PA0495		Allophanate hydrolase		2.72	1.72E-08
PA0496		Allophanate hydrolase		3.08	8E-24
PA1522	*xdhC*	Xanthine dehydrogenase accessory protein		2.07	3.09E-15
PA2003	*bdhA*	3-Hydroxybutyrate dehydrogenase		2.07	7.56E-15
PA2249	*bkdB*	Branched-chain alpha-keto acid dehydrogenase complex component		2.17	4.46E-14
PA2250	*lpdV*	Branched-chain alpha-keto acid dehydrogenase complex component		2.31	8.19E-16
PA2446	*gcvH2*	Glycine cleavage system protein H		2.14	3.3E-10
PA3415		Probable dihydrolipoamide acetyltransferase		3.76	3.12E-36
PA3416		Pyruvate dehydrogenase E1 component subunit beta		2.9	5.1E-10
PA3417		Pyruvate dehydrogenase E1 component subunit alpha		2.69	5.91E-22
PA3582	*glpK*	Glycerol kinase	GlpR binding site	2.23	2.07E-06
PA4792		Putative glycerolphosphodiesterase		2.83	1.42E-41
PA5058	*phaC2*	Poly (3-hydroxyalkanoic acid) synthase; storage polymer polyhydroxyalkanoate (PHA) biosynthesis		2.25	6.21E-16
PA5352	*glcG*	Hypothetical protein		2.02	0.000309
PA5353	*glcF*	Glycolate oxidase iron-sulfur subunit		2.53	2.42E-15
PA5354	*glcE*	Glycolate oxidase FAD-binding subunit		2.49	3.83E-14
PA5355	*glcD*	Glycolate oxidase subunit		2.02	0.0000669

Iron response					
PA0471	*fiuR*	Anti-sigma factor	Fur box	2.13	0.000138
PA0472	*fiuI*	ECF sigma factor; ferric uptake	Fur box	2.01	6.87E-05
PA1302	*hxuA*	TonB-dependent receptor; heme receptor		66.49	0
PA2384	*fur2*	Fur homologue		2.89	2E-07
PA2398	*fpvA*	TonB-dependent receptor; ferripyoverdine receptor	PvdS binding site	2.3	0.000112
PA2403	*fpvG*	Iron dissociation from pyoverdine	PvdS binding site	9.37	2.4E-29
PA2404	*fpvH*	Iron dissociation from pyoverdine	PvdS binding site	14.2	8.67E-71
PA2405	*fpvJ*	Iron dissociation from pyoverdine	PvdS binding site	9.23	1.32E-29
PA2406	*fpvK*	Iron dissociation from pyoverdine	PvdS binding site	10.16	4.65E-38
PA2407	*fpvC*	Periplasmic binding protein		5.52	7.38E-15
PA2408	*fpvD*	ABC transporter ATPase		5.65	2.14E-15
PA2409	*fpvE*	ABC transporter permease		5.98	5.47E-17
PA2410	*fpvF*	Periplasmic binding protein		4.99	4.89E-18
PA2467	*foxR*	Anti-sigma factor FoxR	Fur box	2	2.52E-05
PA3530	*bfd*	Bacterioferritin-associated ferredoxin	Fur box	2.26	3.94E-05
PA4688	*hitB*	Iron (III)-transporter permease		2.09	4.86E-12

Biofilm					
PA1875	*opmL*	Type I toxin efflux outer membrane protein	AmrZ binding site	2.3	4.39E-05
PA2662		Membrane protein		2.36	1.99E-06
PA4293	*pprA*	Two-component sensor; regulation of membrane permeability and *cupE*		2.57	2.18E-17
PA4298		Assembly of type IVb pili	AmrZ/LasR binding site	2.06	0.000718
PA4648	*cupE1*	Fimbriae assembly		2.16	0.000132
PA4651	*cupE4*	Fimbriae assembly		2.22	4.65E-05
PA4675	*chtA*	TonB-dependent receptor; biofilm extracellular matrix		2.15	2.5E-13

DNA damage response (pyocin- and cell lysis-related genes)					
PA0646		F-type pyocin tail fiber protein		2	2.42E-17
PA0807	*ampDh3*	Peptidoglycan hydrolase, cell wall-targeting H2-T6SS effector, AlpA regulon	AmrZ binding site	3.2	1.49E-45
PA0808		Auto-immunity protein for AmpDh3, AlpA regulon		2.17	5.16E-12
PA0819		Hypothetical protein, AlpA regulon	PvdS binding site	2.25	5.69E-05
PA0910	*alpD*	Self-lysis, AlpA regulon		2.09	2.38E-12
PA0911	*alpE*	Self-lysis, AlpA regulon		2.18	1.02E-11
PA0985	*pyoS5*	S-type pyocin		2	8.87E-13

Virulence factors					
PA1871	*lasA*	Protease LasA, staphylolysin		2.37	2.92E-15
PA1899	*phzA2*	Phenazine biosynthesis protein PhzA	AmrZ binding site	3.07	2.79E-31
PA1900	*phzB2*	Phenazine biosynthesis protein PhzB	AmrZ binding site	2.47	9.82E-17
PA1905	*phzG2*	Pyridoxamine 5′-phosphate oxidase	AmrZ binding site	2.58	0.00000117
PA1927	*metE*	5-Methyltetrahydropteroyltriglutamate-homocysteine S-methyltransferase		4.09	4.19E-13
PA3361	*lecB*	Fucose-binding lectin PA-IIL	Lux box	2.31	8.99E-14

Others					
PA0492		Hypothetical protein		2.06	0.000583
PA1887		Hypothetical protein		3.34	6.76E-24
PA1888		Hypothetical protein		2.79	7.61E-23
PA2534		Transcriptional regulator		2.33	4.56E-19
PA2927		Hypothetical protein		3.92	1.28E-54
PA3721	*nalC*	Repressor of MexAB-OprM efflux		2.52	1.77E-24
PA4371		Hypothetical protein		2.08	0.000157
PA5023		Hypothetical protein		2.2	0.000102
PA5446		Hypothetical protein		2	9.77E-13

a PAO1/pMMB-*hxuI* versus PAO1/pMMB with 1 mM IPTG. RNA-seq data were generated by three biological replicates.

As expected, the expression of TonB-dependent transducer *hxuA* was considerably increased (66.5-fold) in the *hxuI* overexpressor (Fig. 3B and Table 1). Beyond that, seven clusters of genes were remarkably upregulated in the *hxuI*-overexpressing strain (Fig. 3B). The upregulated genes, listed here in order from high to low, included the following: (i) the *fpv* gene cluster (PA2403-PA2410), which is involved in iron uptake via siderophore pyoverdine; (ii) the *nir* (PA0509-PA0522), *nor* (PA0523-PA0525), and (iii) *nos* (PA3391-PA3396) gene clusters, which are involved in denitrification of anaerobic respiration; (iv) the PA3415-PA3417 operon encoding putative pyruvate dehydrogenase complex (PDC) which converts pyruvate into acetyl-CoA; (v) the pyocyanin biosynthesis operon *phz2* (PA1899-PA1905); (vi) the *glc* operon (PA5352-PA5355) associated with glycolate utilization and glyoxylate shunt; and (vii) two genes belonging to the *cupE* gene cluster (PA4648-PA4653) which encode fimbriae assembly that promotes biofilm formation (Fig. 3B). To further confirm the transcriptional activation effects of HxuI on the above genes, the promoter regions upstream of *nirS*, *norC*, *phzA2*, *fpvG*, *nosR*, PA3417 (PDC gene), *cupE1*, and *glcD* were fused to a *lacZ* reporter gene and introduced into a PAO1 strain harboring the *hxuI* overexpression plasmid pMMB-*hxuI*. Induction of *hxuI* expression by IPTG resulted in marked increases (7- to 18-fold) in β-galactosidase activity in P*_fpv_*-*lacZ*, P*_nir_*-*lacZ*, P*_nos_*-*lacZ*, and P*_phz2_*-*lacZ* fusions, and modest but significant increases in P*_nor_*-*lacZ*, P*_glc_*-*lacZ*, and P*_cupE_*-*lacZ* fusions (Fig. 3C).

Consistent with the above results, we further observed that (i) pyoverdine production in the *hxuI*-overexpressing strain was significantly higher than that of the wt strain during late exponential phases (Fig. 3D); (ii) under anaerobic condition, the growth rate of the *hxuI* deletion mutant was slower than that of the parent strain PAO1, while no growth defect was observed under aerobic conditions (Fig. 3E); and (iii) booming pyocyanin production was observed in PAO1 which overexpressed *hxuI* (Fig. 3F). In P. aeruginosa, a pair of tandem small RNAs, PrrF1 and PrrF2, promote the production of Pseudomonas quinolone signal (PQS), which activates pyocyanin production (20). In a *prrF1*,*2* double mutant strain background, the activation of pyocyanin production by HxuI disappeared (Fig. 3F), indicating that HxuI-mediated activation of pyocyanin production requires the PrrF small RNAs.

### PA2384 (Fur2) plays a major role in the regulation of *hxuI* regulon.

HxuI was classified as an iron-responsive ECFσ in previous studies, as its promoter region carries a Fur box (21). The ferric uptake regulator (Fur) plays a central role in iron response and is an essential gene in P. aeruginosa (22). The Fur protein employs Fe(II) as a cofactor and binds to a so-called “Fur box” in the promoters of iron-regulated genes, resulting in repression of the target genes; under low-iron conditions, the Fur protein is released from the operator sites and transcription takes place (21). Interestingly, RNA-seq data analysis showed that PA2384 encoding a Fur homologue (designated Fur2) was upregulated 2.89-fold in the PAO1 overexpressing HxuI (Table 1). A HxuI-mediated transcriptional activation was observed in P*_fur2_-lacZ* reporter with a 12-fold increase in β-galactosidase activity (Fig. 3C). Fur2 shares 35% amino acid identity with the N-terminal DNA-binding domain of Fur (PA4764), but does not bear the C-terminal domain of Fur which is responsible for iron binding and dimerization (23). To determine whether Fur2 is involved in the regulation of *hxuI* regulon, we examined the transcriptional activation effects of HxuI on *fpv*, *nir*, *nos*, and *phz2* promoters in a Δ*fur2* mutant. Overexpression of HxuI in the PAO1 strain led to significant increases in β-galactosidase activity in P*_fpv_-lacZ*, P*_nir_-lacZ*, P*_nos_-lacZ*, and P*_phz2_-lacZ* fusions in the wild-type strain (Fig. 3C); however, these HxuI-mediated activations were diminished in the Δ*fur2* mutant background (Fig. S2), suggesting that HxuI-mediated activation of the *fpv*, *nir*, *nos*, and *phz2* genes requires the presence of Fur2. Similarly, overexpression of *hxuI* resulted in 5-fold increases in β-galactosidase activity in PAO1 harboring P*_hxuI_-lacZ* fusion reporter, but not in the *fur2* mutant background (Fig. 3G), indicating that Fur2 is also required for HxuI self-regulation.

### HxuI activates pyocin and bacterial cell lysis-related genes.

**FIG 4:**
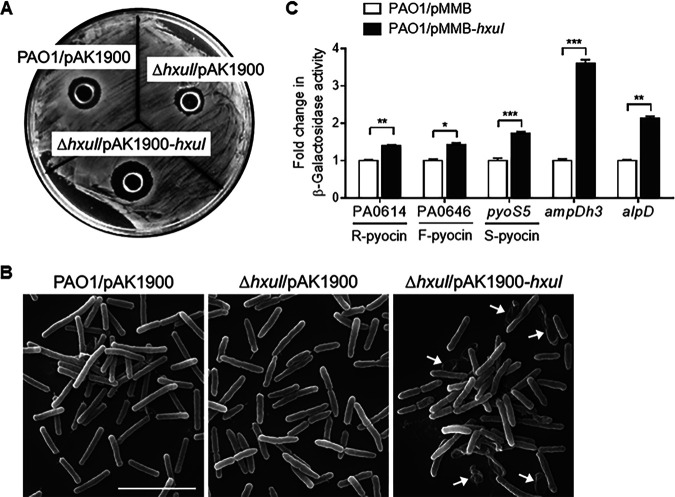
HxuI activates pyocin- and cell lysis-related genes. (A) Zones of clearance in P. aeruginosa PAK strain after exposure to the supernatant of wt PAO1*/*pAK1900 (empty vector), Δ*hxuI/*pAK1900, or Δ*hxuI*/pAK1900-*hxuI* (overexpress *hxuI*). (B) Scanning electron microscopy (SEM) of PAO1 and either Δ*hxuI* containing vector pAK1900 or Δ*hxuI* containing pAK1900-*hxuI*. The scale bar is 5 μm. (C) Promoter-*lacZ* fusions assay. P. aeruginosa PAO1 cells containing the *lacZ* reporter fusions in pDN19 and either plasmid pMMB (empty plasmid) or pMMB-*hxuI* were grown in LB with 1 mM IPTG until late exponential growth phase and analyzed for β-galactosidase activity. Error bars represent SD. *, *P* < 0.05; **, *P* < 0.01; ***, *P* < 0.001.

To establish infection, bacteria must establish a strong foothold for colony development and also outcompete resident microbes. One strategy that potentially addresses both needs is the use of phage tail-like bacteriocins, which are broadly called pyocins in P. aeruginosa (24). Pyocins are released into the environment through explosive cell lysis which kills the producer and nearby competitor bacteria (25). This event also releases extracellular DNA which structurally supports biofilm formation (26). Looking at the RNA-seq data, we noticed that the whole gene sets encoding all three types of pyocins in P. aeruginosa were upregulated in the HxuI-overexpressing strain (Table S1), including the soluble S-type pyocin S2, S4, S5 (PA0985 in Table 1), the contractile R-type pyocin, and the noncontractile F-type pyocin (PA0646 in Table 1). To test whether HxuI is involved in pyocin production, neat supernatants from wt PAO1, Δ*hxuI*, and the complemented strain Δ*hxuI*/pAK1900-*hxuI* were spotted onto an L agar overlay containing the indicator P. aeruginosa strain PAK. As shown in Fig. 4A, the growth inhibition zone of the Δ*hxuI*/pAK1900*-hxuI* strain was larger than that of the wt and the Δ*hxuI* mutant, indicating higher intraspecies competitiveness that might be mediated by pyocin production. In addition, two sets of cell lysis genes, PA0807 (*ampDh3*)-PA0808 (immunity of AmpDh3) and *alpDE* (27), were upregulated at average rates of ∼2.7-fold and ∼2.1-fold, respectively, in the HxuI overexpressor (Table 1). AmpDh3, a cell wall amidase, is thought to be delivered by the type VI secretion system locus II (H2-T6SS) to bacterial competitors and degrade the cell wall peptidoglycan of prey, thereby providing a growth advantage for P. aeruginosa (28). AlpDE belongs to the AlpBCDE self-lysis cassette which responds to DNA damage inflicted by the host immune system and enhances the virulence of P. aeruginosa (29). Under scanning electron microscopy (SEM), more bacterial cell lysis was observable in the Δ*hxuI*/pAK1900*-hxuI* culture than in the wt strain culture (Fig. 4B); cells that overexpressed *hxuI* were inclined to gather together on the coverslips and form colony-like architectures, while Δ*hxuI* cells were scattered evenly (Fig. 4B). To investigate the transcriptional activation effects of HxuI on the above genes, the promoter regions upstream of PA0614 (R-pyocin), PA0646 (F-pyocin), *pyoS5*, *ampDh3*, and *alpD* were fused to the *lacZ* reporter and introduced into a PAO1 strain harboring the plasmid pMMB-*hxuI*. Significant increases in β-galactosidase activity were observed in all five fusions when HxuI expression was induced (Fig. 4C).

### DNA recognition sites of HxuI.

**FIG 5:**
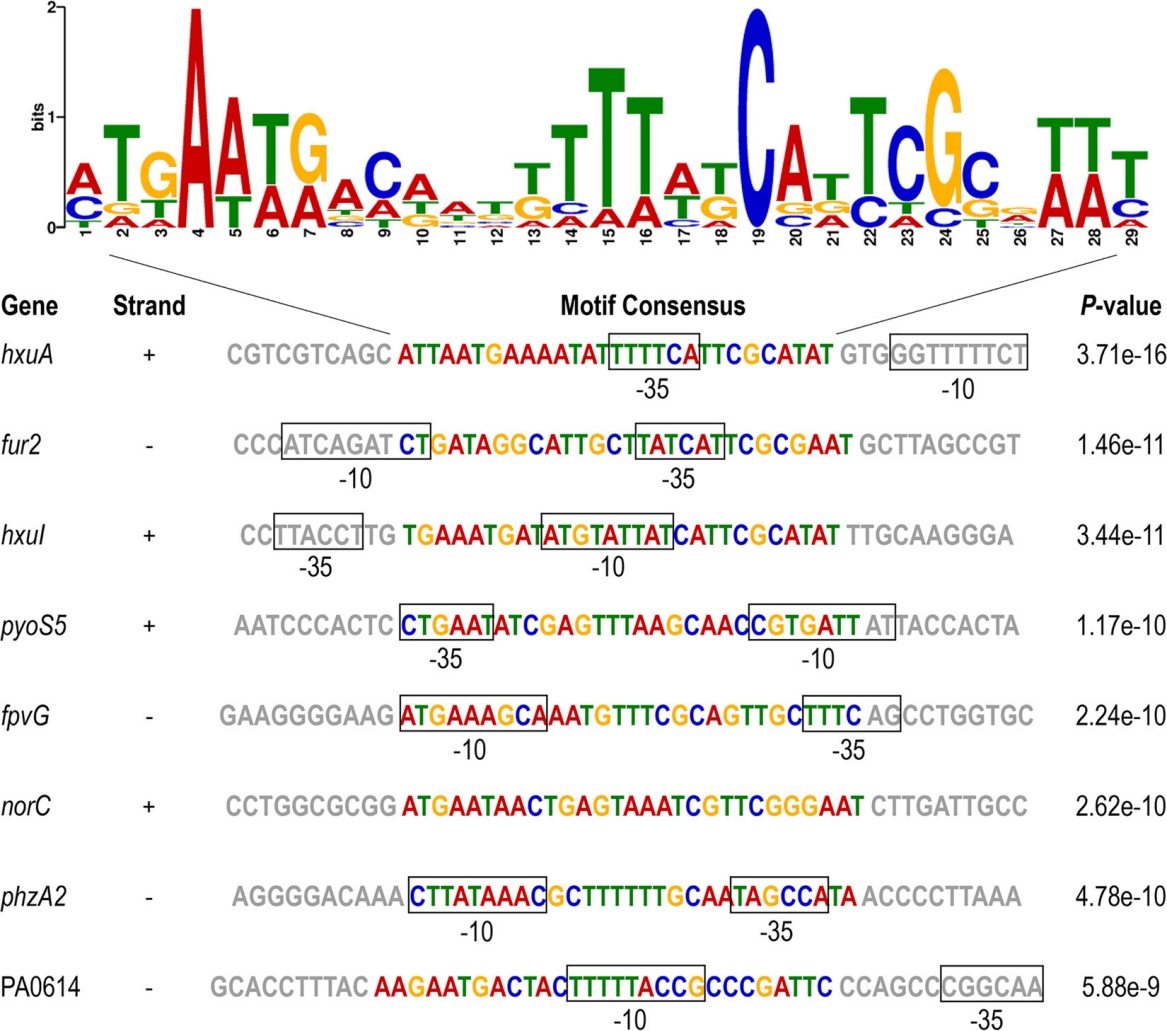
HxuI recognition motif predicted by MEME. The positions of the -35 and -10 boxes in promoter DNAs are predicted by the BPROM online service (46). The potential promoter region of *norC* is not included in the indicated sequence.

To accurately redirect gene expression, ECFσ select promoters with high stringency by combining sequence-specific interactions with the -10 and -35 promoter elements (10). To identify the specific DNA sequences that are recognized by HxuI, we analyzed the HxuI binding motif by using the MEME online tool (http://memesuite.org/tools/meme) (30) on the promoter regions of *fpvG*, *nirS*, *norC*, *nosR*, *phzA2*, *glcD*, *cupE1*, *fur2*, PA0614, PA0646, *pyoS5*, *ampDh3*, *alpD*, *hxuA*, and *hxuI*. The MEME analysis revealed a consensus motif of 5′-MTGAAWRACDWKKTTTWKCADTCGCRWWT-3′ as the potential HxuI binding site (Fig. 5). The genes *hxuA*, *fur2*, *pyoS5*, *fpvG*, *phzA2*, and PA0614 carry this motif in their promoter regions (Fig. 5), hinting these genes may be the direct targets of HxuI.

### HxuI promotes P. aeruginosa infection in mice.

**FIG 6:**
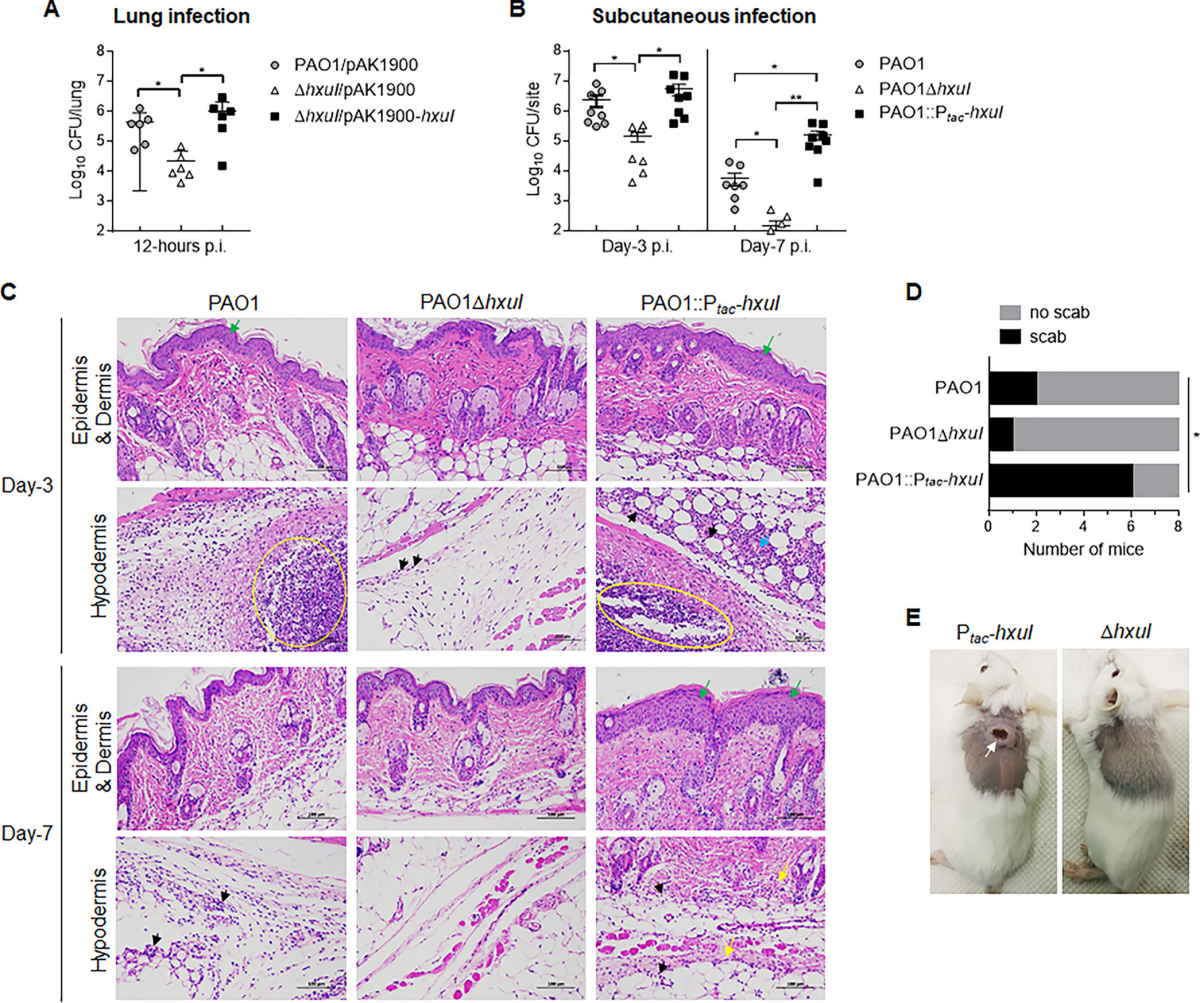
HxuI promotes P. aeruginosa infection in murine models. (A) In the acute pneumonia model, mice (*n* = 6/group) were intranasally inoculated with 1 × 10^7^ CFU of the indicated bacterial cells. Bacterial loads in lungs were counted by plating at 12 h postinfection (pi). (B) In the cutaneous abscess model, mice (*n* = 8/group) were subcutaneously inoculated with 5 × 10^6^ CFU of indicated bacterial cells. Bacterial loads in abscesses were counted on days 3 and 7 pi. Error bars represent SD. *, *P* < 0.05; **, *P* < 0.01. (C) Histological sections of cutaneous abscess. Yellow circles indicate inflammation and tissue injury, green arrows indicate thickening of the epidermis, black arrows indicate neutrophil infiltration, blue arrow indicates extravasated blood in capillaries, yellow arrows indicate fiber necrosis. (D) Scab formation on day 7 pi. *P* value was calculated using one-way ANOVA; *, *P* < 0.05. (E) Skin appearance of scab (white arrow) on day 7.

A mouse lung infection model was used to determine the role of HxuI in acute infection. Mice were intranasally infected with the same amount of wt PAO1, Δ*hxuI* mutant, and *hxuI* complementary strain, respectively. At 12 h postinfection, the *hxuI* deletion mutant exhibited a significantly lower bacterial load in lungs compared to that of wt PAO1, and complementation with *hxuI* restored bacterial colonization capacity to wt levels (Fig. 6A). These data indicated that HxuI is critical for colonization in P. aeruginosa. A murine cutaneous abscess model was further employed as a chronic infection model (31) to determine the role of HxuI in long-term infection. To avoid the loss of HxuI expression vector, *hxuI* driven by *tac* promoter was inserted into the PAO1 chromosome via a mini-Tn7 vector (PAO1::*P_tac_-hxuI*), resulting in a constitutive expression of the *hxuI* gene (32, 33). Mice were subcutaneously inoculated with 5 × 10^6^ CFU of wt PAO1, Δ*hxuI*, or PAO1::P*_tac_-hxuI*. On day 3 postinfection, the Δ*hxuI* mutant-infected group exhibited a lower bacterial burden in lesions than those infected by wt PAO1 or PAO1::P*_tac_-hxuI* (Fig. 6B). Histological examinations of skin abscesses indicated intense inflammatory infiltration, local tissue necrosis, and thickening of the epidermis in both PAO1 and PAO1::P*_tac_-hxuI* infection groups, while infection by Δ*hxuI* resulted in very mild inflammations (Fig. 6C). On day 7, a large abscess with overlying crust/scab was formed on the dorsum skin of 75% (6/8) mice infected by PAO1::P*_tac_-hxuI*, but on only 25% (2/8) and 12.5% (1/8) of those infected by PAO1 and Δ*hxuI*, respectively (Fig. 6D and E). Histological sections of the PAO1::P*_tac_-hxuI*-infected group showed thickened epidermis, collagen fiber necrosis, lysis of subcutaneous muscle fibers, and inflammation (Fig. 6C). In comparison, the PAO1 and Δ*hxuI* infection groups exhibited much lower bacterial loads inside abscesses and fewer scattered inflammatory cells (Fig. 6B and C). These results indicated that forced expression of the HxuI enables P. aeruginosa to better adapt to the host environment, promoting the establishment of long-term infection.

## DISCUSSION

In this study, we found that ECFσ factor HxuI is highly conserved in different P. aeruginosa strains and can be induced by several host-inflicted stresses, including iron deprivation, oxidative stress, and hypoxia, as well as NO. Physiological adaptation to varied environmental stresses, such as changes in oxygen levels encountered within diverse niches, is an important capability for pathogenic bacterial species (34). The viability of P. aeruginosa within robust anaerobic biofilms requires NO reductase to modulate or prevent the accumulation of toxic NO, a byproduct of anaerobic respiration (35). Our data indicate that the NO sensor DNR negatively regulates HxuI, which further activates denitrification to reduce NO into nitrogen gas (36), revealing a novel ECFσ-mediated nitrosative stress-response pathway in P. aeruginosa.

Overexpression of HxuI remarkably activated the transcription of genes associated with pyoverdine-dependent iron acquisition, denitrification, pyocyanin biosynthesis, and the production of pyocins involved in intraspecies competition. Fur2 is positively regulated by HxuI and plays a critical role in HxuI-mediated transcriptional regulation, and even in the auto-activation of HxuI. Most notably, forced expression of the *hxuI* gene promotes the establishment of long-term P. aeruginosa infection *in vivo*; therefore, HxuI functions as an important regulator that senses host stresses and enables P. aeruginosa to tune metabolic strategies for adaptation to the host environment and express virulence factors which promote persistent infection.

## MATERIALS AND METHODS

### Bacterial strains, plasmids, and growth conditions.

The bacterial strains, plasmids, and primers used in this study are listed in Table S2. Gene deletion and complementation were performed as previously described (37, 38). Bacterial cells were grown at 37°C in LB (Luria-Bertani) broth or in M9 medium with 0.1% (wt/vol) glucose (39). The following concentrations of antibiotics were used: for P. aeruginosa, gentamicin at 30 μg/mL in LB, tetracycline at 50 μg/mL in LB, and carbenicillin at 150 μg/mL in LB; for Escherichia coli, tetracycline at 10 μg/mL, gentamicin at 10 μg/mL, kanamycin at 50 μg/mL, and ampicillin at 100 μg/mL. For iron-limitation condition, strains were grown in ABTG medium [15.1 mM (NH4)_2_SO_4_, 33.7 mM Na_2_HPO_4_, 22.0 mM KH_2_PO_4_, 0.05 mM NaCl, 1 mM MgCl_2_, 100 μM CaCl_2_, 0.5% (wt/vol) glucose, and 1 to 10 μM FeCl_3_] (40). Anaerobic conditions were established by an anaerobic workstation (Don Whitley Scientific) with an oxygen content of 0.07%, and bacteria were statically cultured in various media supplemented with 50 mM NaNO_3_. All experiments were done in the biosafety level 2 laboratory at Nankai University.

### Gene conservation analysis.

The population structure of P. aeruginosa can be divided into five groups (41). The complete genomes of 723 P. aeruginosa strains that covered all five groups were analyzed in this study. The nucleotide sequences of the *huxIRA* of PAO1 were used as reference. We aligned each genome sequence of the 723 strains against the reference using BLASTn (14) with the criteria set as E value < 1e-5 and length coverage of the gene > 85% to find the homologous sequences. Finally, the identities between each strain and reference were illustrated using the R package (http://www.r-project.org/).

### Ethics statement.

All animal studies complied with National and Nankai University guidelines regarding the use of animals in research. All animal experiment protocols were approved by the Institutional Animal Care and Use Committee of the College of Life Sciences of Nankai University with the permit number NK-04-2012.

### Murine lung infection.

The infection of mice was performed as previously described (42). Briefly, overnight bacterial culture was diluted 1:100 in fresh LB and grown at 37°C until the OD_600_ reached 1.0. Bacterial cells were collected by centrifugation and washed once with phosphate-buffered saline (PBS). The bacterial cell concentration was adjusted to 5 × 10^8^ CFU/mL in PBS. Each female BALB/c mouse (Vital River, Beijing, China), at the age of 6 to 8 weeks, was anesthetized with an intraperitoneal injection of 7.5% chloral hydrate and inoculated with 20 μL of the bacterial suspension, resulting in 1 × 10^7^ CFU per mouse. Bronchi alveolar lavage fluid (BALF) was collected as previously described (43). At 6 h postinfection, mice were euthanized via CO_2_ inhalation. One mL PBS containing 0.05 mM EDTA was injected into the lungs via the trachea by a vein detained needle (BD, Angiocath). After 1 min of detaining, BALF was collected.

### Total RNA isolation and quantitative real-time PCR.

Total bacterial RNA was isolated using an RNAprep Pure Cell/Bacteria Kit (Tiangen Biotec, Beijing, China). cDNAs were synthesized with reverse transcriptase and random primers (Takara Bio, Dalian, China). Real-time (RT) PCR was performed using SYBR II Green Supermix (Bio-Rad, Beijing, China). Specific Primers (Table S3) were used for quantitative RT-PCR. The peptidyl-prolyl *cis*-*trans* isomerase D gene *ppiD* was used as an internal control.

### Transcriptome sequencing and data analysis.

Both PAO1/pMMB and PAO1/pMMB-*hxuI* cultures (OD_600_=0.6) were grown in LB with 1 mM IPTG for 2 h. Total RNA was isolated using an RNAprep Pure Cell/Bacteria Kit (Tiangen Biotec, Beijing, China). Three replicates were prepared for each strain. Sequencing and analysis were performed as previously described (44).

### Promoter-*lacZ* reporter assay.

The promoter region (500 bp upstream from the start codon) of each gene was cloned into pDN19*lac*Ω to construct the promoter-*lacZ* reporter construct. The reporter constructs, as well as the pMMB-*hxuI* or the empty plasmid pMMB, were introduced into PAO1 by electroporation, and the transformants were selected on an L agar plate containing Tc and Cb. After inducing the expression of HxuI with 1 mM IPTG for 2 h, bacterial cells were collected by centrifugation and resuspended in 500 μL of Z-buffer (16 g/L Na_2_HPO_4_·7 H_2_O, 4.8 g/L NaH_2_PO_4_, 0.746 g/L KCl, 0.246 g/L MgSO_4_·7 H_2_O, 3.5 mL/L β-mercaptoethanol [pH = 7]). To permeabilize the cells, 10 μL of 0.1% SDS and 10 μL of chloroform were added and vortex for 10 s. After this, 100 μL of 4 mg/mL ONPG (*o*-nitrophenyl-β-d-galactopyranoside) was added to the cells. The samples were incubated at 37°C until the yellow color became apparent, and 500 μL of Na_2_CO_3_ (0.5 M) was added to stop the reaction. Sample absorbance was read at 420 nm, and β-galactosidase activity was calculated as Miller units = 2,000 × OD_420_/OD_600_/incubation time (min). Each assay was repeated three times.

### Measurement of pyoverdine production.

A microplate pyoverdine measurement was carried out in ABTGC medium [15.1 mM (NH4)_2_SO_4_, 33.7 mM Na_2_HPO_4_, 22.0 mM KH_2_PO_4_, 0.05 mM NaCl, 1 mM MgCl_2_, 100 μM CaCl_2_, 10 μM FeCl_3_, 0.2% (wt/vol) glucose and 0.2% (wt/vol) casamino acid] as previously described (45). The overnight P. aeruginosa cultures were adjusted to an OD_600_ of 0.01 in ABTGC medium. The cells were then incubated in 96-well plates at 37°C. Pyoverdine fluorescence (excitation maximum 400 nm, emission maximum 460 nm) and OD_600_ were recorded by the microplate reader (Tecan Group Ltd., Switzerland) every hour. Experiments were performed in triplicate, and results are shown as the mean ± SD (standard deviation).

### Pyocin toxicity assays.

Zones of clearance were observed for the P. aeruginosa PAK strain using the supernatants of wt PAO1, *hxuI* mutant and *hxuI*-overexpressing strains. A 0.05 μg/mL volume of ciprofloxacin was used to induce the production of pyocins in the PAO1-derived strains. PAK was used as an indicator strain, diluted to OD_600_ = 0.6, and plated on LB agar. Finally, 200 μL of supernatants of the test strains were added to sterile Oxford cups placed on the PAK plate and cultured overnight at 37°C.

### Scanning electron microscopy (SEM).

Bacterial cultures (OD_600_ = 1.0) were co-incubated with 0.1% gelatin-coated glass slides at 37°C for 4 h. The unattached bacterial cells were discarded. The glass slides with sessile bacteria were washed once with PBS and fixed with 4% paraformaldehyde. The bacterial cells were dehydrated with a gradient (30%, 50%, 70%, 90%, 100%) of alcohol, air dried, and imaged under an electron microscope.

### Mouse cutaneous abscess model.

The infection of mice was performed as previously described (31). Briefly, mice were clipped in the dorsal area by a shaver and depilatory cream. Fifty μL of either 5 × 10^6^ CFU bacterial suspension or saline were subcutaneously injected into the dorsum of each mouse. At 3 and 7 days postinfection, mice were euthanized with carbon dioxide, and then the skin abscesses were excised, homogenized in saline, and subjected to plating for CFU counting.

### Statistical analysis.

Statistical evaluations were performed using GraphPad Prism 7.0 (GraphPad Software Inc., La Jolla, CA). *P* values were calculated using one-way analysis of variance (ANOVA), a two-tailed unpaired Student’s *t* test. Data were considered significant when *P* values were below 0.05, as indicated.

### Data availability.

The transcriptome (RNA-Seq) data have been deposited in NCBI BioProject with the accession code PRJNA717102.
