# Supplementary material for: ECF Sigma Factor HxuI Is Critical for In Vivo Fitness of Pseudomonas aeruginosa during Infection
Source: Microbiol Spectr. 2022 Jan 19;10(1):e01620-21. doi: 10.1128/spectrum.01620-21 (PMC8768829; doi:10.1128/spectrum.01620-21)
Supplement: SUPPLEMENTAL FILE 1 — Supplemental material. Download SPECTRUM01620-21_Supp_1_seq9.pdf, PDF file, 0.3 MB [file spectrum01620-21_supp_1_seq9.pdf]

Supplemental Information

ECF sigma factor HxuI is critical for *in vivo* fitness of *Pseudomonas aeruginosa* during infection

Zeqiong Cai[1], Fan Yang[1], Xiaolong Shao[2], Zhuo Yue[1], Zhenpeng Li[3], Yuqin Song[4], Xiaolei Pan[1], Yongxin Jin[1], Zhihui Cheng[1], Un-Hwan Ha[5], Jie Feng[4], Liang Yang[6], Xin Deng[2], Weihui Wu[1], Fang Bai[1]*

[1] State Key Laboratory of Medicinal Chemical Biology, Key Laboratory of Molecular Microbiology and Technology of the Ministry of Education, College of Life Sciences, Nankai University, Tianjin, 300071, China.

[2] Department of Biomedical Sciences, City University of Hong Kong, Kowloon Tong, Hong Kong SAR, China.

[3] School of Laboratory Medicine, Key Laboratory of Clinical Laboratory Diagnostics in Universities of Shandong, Weifang Medical University, 261053 Weifang, Shandong, China.

[4] State Key Laboratory of Microbial Resources, Institute of Microbiology, Chinese Academy of Sciences, Beijing 100101, China.

[5] Department of Biotechnology and Bioinformatics, Korea University, Sejong 30019, Republic of Korea.

[6] School of Medicine, Southern University of Science and Technology (SUSTec), Shenzhen 518055, China.

*, Corresponding authors: Fang Bai (baifang1122@nankai.edu.cn)

This file contains

1) **Supplementary Figure 1-2.**
2) **Supplementary Table 1-3.**
3) **Reference.**

Supplementary Figures

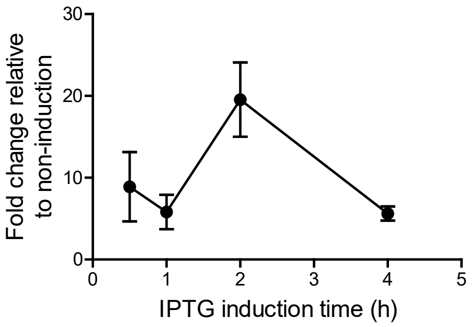

Figure S1. Expression level of *hxuA* (PA1302) in PAO1/pMMB-*hxuI* following induction by 1 mM IPTG, determined by q-PCR at indicated time points.

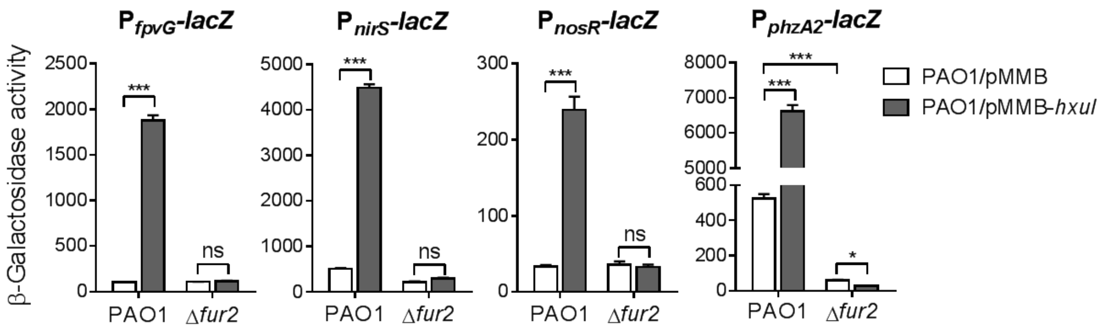

Figure S2. Analysis of the promoter-*lacZ* receptor expression. *P. aeruginosa* PAO1 or PAO1Δ*fur2* containing the indicated *lacZ* reporter fusions and pMMB or pMMB-*hxuI* were grown in LB with 1 mM IPTG until late exponential growth phase and analyzed for β-galactosidase activity. Error bars represent SD. *$P < 0.05$, ***$P < 0.001$; ns, not significant.

Supplementary Tables

Table S1. Upregulated pyocin genes in *P. aeruginosa* PAO1 that overexpressing HxuI [a].

locus_tag	Gene	Describes	Fold Change[a]	*P*-value
S-type pyocin				
PA1150	*pys2*	pyocin S2	1.85	1.53E-17
PA3866		pyocin S4	1.67	2.06E-12
PA0985	*pyoS5*	pyocin S5	2.00	8.87E-13
R-type pyocin				
PA0614		pyocin R2, holin	1.68	3.57E-08
PA0615		phage protein	1.90	5.31E-11
PA0616		phage baseplate assembly protein V	1.53	2.35E-06

PA0617		phage baseplate assembly protein W	1.62	2.72E-06
PA0618		phage baseplate assembly protein J	1.75	2.22E-14
PA0619		phage tail protein I	1.73	2.06E-12
PA0620		phage tail fiber protein H	1.67	4.84E-11
PA0621		tail fiber assembly protein	1.67	8.80E-10
PA0622		phage tail sheath protein	1.91	4.06E-14
PA0623		pyocin R2 tail tube protein	1.86	4.30E-15
PA0624		hypothetical protein	1.73	8.49E-09
PA0625		pyocin R2 tail length determination protein	1.85	2.33E-12
PA0626		pyocin R2 tail formation protein	1.79	2.05E-12
PA0627		pyocin R2 tail component protein X	1.51	0.0108608
PA0628		phage late control protein D	1.78	2.31E-10
PA0629		pyocin R2 lytic enzyme	1.63	9.23E-08
F-type pyocin				
PA0633		major tail protein	1.92	9.02E-15
PA0634		hypothetical protein	1.71	1.36E-06
PA0635		phage protein	1.90	1.45E-08
PA0636		tail tape measure protein	1.96	4.44E-15
PA0637		phage minor tail protein	1.75	1.56E-07
PA0638		phage minor tail protein L	1.99	3.41E-17
PA0639		phage tail assembly protein K	1.82	6.69E-15
PA0640		phage tail assembly protein I	1.78	3.74E-12
PA0641		phage-related protein, tail component	1.79	1.95E-14
PA0643		phage tail fiber protein	1.82	1.94E-14
PA0644		hypothetical protein	1.52	0.0499101
PA0645		hypothetical protein	1.61	0.0004555
PA0646		tail fiber protein	2.00	2.42E-17
PA0647		hypothetical protein	1.74	0.0001786
PA0648		hypothetical protein	1.63	1.13E-08

[a] RNA-seq identifies fold change in gene expression of PAO1/pMMB-*hxuI* versus PAO1/pMMB after 2 hours of induction with 1 mM IPTG.

Table S2. Bacterial strains and plasmids used in this study.

**Strains/plasmids**	**Description**	**Source**
P. aeruginosa		
PAO1	Laboratory standard strain; wild-type (wt)	Lab stock
PAO1Δ*hxuI*	*hxuI* deletion mutant of PAO1	This study
PAO1::P_{tac}-*hxuI*	*hxuI* driven by *tac* promoter was inserted into the PAO1 chromosome by a mini-Tn7 vector	This study
PAO1Δ*oxyR*	*oxyR* deletion mutant of PAO1	This study
PAO1Δ*oxyR*/C	Complemented strain with *oxyR* driven by self-promoter, which was chromosomally inserted by mini-Tn7	This study

PAO1Δ*anr*	*anr* deletion mutant of PAO1	This study
PAO1Δ*dnr*	*dnr* deletion mutant of PAO1	This study
PAO1Δ*dnr*/C	Complemented strain with *dnr* driven by self-promoter, which was chromosomally inserted by mini-Tn7	This study
PAO1Δ*fur2*	*fur2* (PA2384) deletion mutant of PAO1	This study
E. coli		
DH5α	For general cloning and sub-cloning; *lacZ*ΔM15, *recA1*, *endA1*	Lab stock
S17	Donor strain for conjugation	Lab stock
Plasmids		
pMMB67EH	Inducible expression vector with *tac* promoter, Ap^r, Cb^r	(1)
pMMB67EH-*hxuI*	Overexpression of *hxuI* on pMMB67EH	This study
pAK1900	Expression vector with *lac* promoter, Ap^r, Cb^r	(2)
pAK1900-*hxuI*	Overexpression of *hxuI* on pAK1900	This study
pEX18Tc	Gene replacement vector, Tc^r, *oriT*$^+$, *sacB*$^+$	(3)
pDN19*lac*Ω	Promoterless *lacZ* transcription fusion vector, Ω fragment, Sp^r, Sm^r, Tc^r	(4)
pUC18T-mini-Tn7T-Gm	mini-Tn7 base vector from insertion into chromosome *att*Tn7 site, Gm^r	(5)

Table S3. Primers used in this study.

**Primer**	**Sequence (5' to 3')**	**Purpose**
q-*hxuI*-F	TATCAGGCGTTCCATGGCG	qRT-PCR
q-*hxuI*-R	CAGCCACTGGGCGAAAGTA	
q-*hxuR*-F	CGAAACCGAAGAGGACATCG	qRT-PCR
q-*hxuR*-R	CGTAGCCAATCCTCCAGCTC	
q-*hxuA*-F	CGTTGTTCCAGAACCGCATC	qRT-PCR
q-*hxuA*-R	TACCACCGATACTGCCGTTG	
q-*ppiD*-F	CGCTGAAGCAAGGTGAGGTA	qRT-PCR
q-*ppiD*-R	GCTTTCCAGGCTCGGTACTT	
hxuI-up-F	GAGCTCGGTACCCGGGGATCCTGAACGGCCTGATGAACGAAGTG	*hxuI* deletion
hxuI-up-R	TCGTTCCAGCGGCTTCATCCTTCCCTGATAGGCATGCGAGACGGAA	
hxuI-down-F	TTCCGTCTCGCATGCCTATCAGGGAAGGATGAAGCCGCTGGAACGA	
hxuI-down-R	ACGACGGCCAGTGCCAAGCTTGATAGCGCGACAGTTCCTCCAC	
fur2-up-F	GAGCTCGGTACCCGGGGATCCTACGCCCTGAGCCGGCTACA	*fur2* deletion
fur2-up-R	GCTGCCCGAGATCAGGGATACACATCTGTTTGACGGGCGAAT	
fur2-down-F	ATTCGCCCGTCAAACAGATGTGTATCCCTGATCTCGGGCAGC	
fur2-down-R	ACGACGGCCAGTGCCAAGCTTGAATTCCCTGCCCTGGACCAC	
anr-up-F	TATGACCATGATTACGAATTCAGACAGAGTTCGGCATCGACTTC	*anr* deletion
anr-up-R	TCGATGGAGTCGAGGATGTGCACCACCTTGATGGTTTCGGCCATT	
anr-down-F	AATGGCCGAAACCATCAAGGTGTGCACATCCTCGACTCCATCGA	
anr-down-R	ACGACGGCCAGTGCCAAGCTTTGGCAGTCATGGCTAACTCCTTT	
dnr-up-F	TATGACCATGATTACGAATTCCCTGGTGCCGTACATGGTCGAA	*dnr* deletion

dnr-up-R	TTCGCGGTCGAGGATGCTGATTAGAAAGCGGTTCGAACAGGTGG	
dnr-down-F	CCACCTGTTCGAACCGCTTTCTAATCAGCATCCTCGACCGCGAA	
dnr-down-R	ACGACGGCCAGTGCCAAGCTTAGCCAGGCCTGTTCCTCGAACA	
oxyR-up-F	TATGACCATGATTACGAATTCTACCGAATGTTCGTCGGCCCATT	*oxyR* deletion
oxyR-up-R	ATTTGCGGTTGTTCCTGGGTTTGACGATGTAGCGCAGTTCGGTGA	
oxyR-down-F	TCACCGAACTGCGCTACATCGTCAAACCCAGGAACAACCGCAAAT	
oxyR-down-R	ACGACGGCCAGTGCCAAGCTTTGGTAACCGGCCTCTATGGCTT	
prrF-up-F	TATGACCATGATTACGAATTCAAGATATTTACTCCCCCCGGGCG	*prrF* deletion
prrF-up-R	GCGGGCAAAAAAAGACCCGGCCTCGCGACCAGTTGAGTGACATA	
prrF-down-F	TATGTCACTCAACTGGTCGCGAGGCCGGGTCTTTTTTTGCCCGC	
prrF-down-R	ACGACGGCCAGTGCCAAGCTTTTGAAGGAGTCGCCATGCGCATT	
HxuI01-F	TTCACACAGGAAACAGAATTCATGGATGAGCGCTTCCGTGGTTC	Overexpression of
HxuI01-R	TCCGCCAAAACAGCCAAGCTTAACAGCGGTTCCAGTCGTTCCAG	*hxuI* on pMMB67EH
HxuI02-F	ACACTATAGAATACTCAAGCTTCCGGAGGATGCACGGATGGAT	Overexpression of
HxuI02-R	GAGCTCGGTACCCGGGGATCCTCCAGTCGTTCCAGCGGCTTC	*hxuI* on pAK1900
P*tac*-*Hin*d3-F	CGAGGTACCGGGCCCAAGCTTAACGTAAATGCCGCTTCGCCT	Construction of P_{tac}-
P*tac*-R	GTTGACCCGCTGCAACACCTCCACACATTATACGAGCCGATG	*hxuI* on mini-Tn7
HxuI04-F	CATCGGCTCGTATAATGTGTGGAGGTGTTGCAGCGGGTCAAC	base vector
HxuI04-*Sac*I-R	AATTCGATCATGCATGAGCTCTTCATCCTTCGCCTCCCTGGC	
*nirS*p-F	TTAAAACGACGGCCAGTGAATTCTTCGTCCAGGTAGCAGATACCGC	*lacZ* promoter assay
*nirS*p-R	TATCTAGAACCTCCTTAGGATCCCATGGTCTATCTCCTCAGGAGCCC	
*nirQ*p-F	TTAAAACGACGGCCAGTGAATTCTGGATGTACTTGGCCATCAGGG	*lacZ* promoter assay
*nirQ*p-R	TATCTAGAACCTCCTTAGGATCCCATGTCCTACTCCTGCGCTAGG	
*phzA2*p-F	TTAAAACGACGGCCAGTGAATTCGCGCACGGTGAAGTTCCATATCG	*lacZ* promoter assay
*phzA2*p-R	TATCTAGAACCTCCTTAGGATCCCATGGTGCGAATCTCCGCCAGTT	
*fpvG*p-F	TTAAAACGACGGCCAGTGAATTCACCGTACGCAGGGTTTCATGAC	*lacZ* promoter assay
*fpvG*p-R	TATCTAGAACCTCCTTAGGATCCCATGAGGCTAACGGTAGGTTAGGG	
*nosR*p-F	TTAAAACGACGGCCAGTGAATTCAACGAAGACCTGGTGTATCTCACC	*lacZ* promoter assay
*nosR*p-R	TATCTAGAACCTCCTTAGGATCCCATAGGTTCCATCTACTACTGGCTA CC	
PA3417p-F	TTAAAACGACGGCCAGTGAATTCATTGCTCTCCACCCCTTGCGA	*lacZ* promoter assay
PA3417p-R	TATCTAGAACCTCCTTAGGATCCCATCGCAAACCTCCTCCGCAC	
*glcD*p-F	TTAAAACGACGGCCAGTGAATTCGACTTCGAGCAGGTCGTAGAGG	*lacZ* promoter assay
*glcD*p-R	TATCTAGAACCTCCTTAGGATCCCATGGCGGCTGTCCTTGTTGT	
*cupE1*p-F	TTAAAACGACGGCCAGTGAATTCATCGTCGCCATCGTCCTCAAC	*lacZ* promoter assay
*cupE1*p-R	TATCTAGAACCTCCTTAGGATCCCATCTCTAACTTCCTGTCGATAACG	
*hxuI*p-F	TTAAAACGACGGCCAGTGAATTCCCTGCAGAAGCTCGAGGACGAG	*lacZ* promoter assay
*hxuI*p-R	TATCTAGAACCTCCTTAGGATCCCATCCGTGCATCCTCCGGCG	
*fur2*p-F	TTAAAACGACGGCCAGTGAATTCGACCTGCAAGTGAGCACCCGTG	*lacZ* promoter assay
*fur2*p-R	TATCTAGAACCTCCTTAGGATCCCATCGTGACGCTCCTTTCGTGG	
PA0614p-F	TTAAAACGACGGCCAGTGAATTCTGGAGCACTATCTCAATCGCGA	*lacZ* promoter assay
PA0614p-R	TATCTAGAACCTCCTTAGGATCCCGAGTGCCTCCCTGGGGACGCAC	
PA0646p-F	TTAAAACGACGGCCAGTGAATTCTGCCTCCGGAGGCATACCATGA	*lacZ* promoter assay

PA0646p-R	TATCTAGAACCTCCTTAGGATCCCGACGTTCTCCAGATAATGAAAAA	
*pyoS5*p-F	TTAAAACGACGGCCAGTGAATTCGCCCGCCAGGTGTAGAACGAGG	*lacZ* promoter assay
*pyoS5*p-R	TATCTAGAACCTCCTTAGGATCCCTTAGACTTCTCCATTGGTGAG	
*ampDh3*p-F	TTAAAACGACGGCCAGTGAATTCGTGAACTTTGTGAAGAAGTGCA	*lacZ* promoter assay
*ampDh3*p-R	TATCTAGAACCTCCTTAGGATCCCGGTTTTCACCTTTTTGCGGATG	
*alpD*p-F	TTAAAACGACGGCCAGTGAATTCACCAGCGGCATGGCCGCCTTCG	*lacZ* promoter assay
*alpD*p-R	TATCTAGAACCTCCTTAGGATCCCGGCGAACTCCTTTTTCAGGATG	

References

1. Fürste JP, Pansegrau W, Frank R, Blöcker H, Scholz P, Bagdasarian M, Lanka E. 1986. Molecular cloning of the plasmid RP4 primase region in a multi-host-range tacP expression vector. Gene 48:119-31.
2. Jansons I, Touchie G, Sharp R, Almquist K, Farinha MA, Lam JS, Kropinski AM. 1994. Deletion and transposon mutagenesis and sequence analysis of the pRO1600 OriR region found in the broad-host-range plasmids of the pQF series. Plasmid 31:265-74.
3. Hoang TT, Karkhoff-Schweizer RR, Kutchma AJ, Schweizer HP. 1998. A broad-host-range Flp-FRT recombination system for site-specific excision of chromosomally-located DNA sequences: application for isolation of unmarked Pseudomonas aeruginosa mutants. Gene 212:77-86.
4. Totten PA, Lory S. 1990. Characterization of the type a flagellin gene from Pseudomonas aeruginosa PAK. J Bacteriol 172:7188-99.
5. Choi K-H, Schweizer HP. 2006. mini-Tn7 insertion in bacteria with single attTn7 sites: example Pseudomonas aeruginosa. Nature Protocols 1:153-161.
